# Real-Time fMRI Neurofeedback Training Changes Brain Degree Centrality and Improves Sleep in Chronic Insomnia Disorder: A Resting-State fMRI Study

**DOI:** 10.3389/fnmol.2022.825286

**Published:** 2022-02-23

**Authors:** Xiaodong Li, Zhonglin Li, Zhi Zou, Xiaolin Wu, Hui Gao, Caiyun Wang, Jing Zhou, Fei Qi, Miao Zhang, Junya He, Xin Qi, Fengshan Yan, Shewei Dou, Hongju Zhang, Li Tong, Yongli Li

**Affiliations:** ^1^Department of Radiology, Henan Provincial People’s Hospital, People’s Hospital of Zhengzhou University, Zhengzhou, China; ^2^Department of Nuclear Medicine, Henan Provincial People’s Hospital, People’s Hospital of Zhengzhou University, Zhengzhou, China; ^3^Henan Key Laboratory of Imaging and Intelligent Processing, PLA Strategic Support Force Information Engineering University, Zhengzhou, China; ^4^Health Management Center, Henan Provincial People’s Hospital, People’s Hospital of Zhengzhou University, Zhengzhou, China; ^5^Department of Neurology, Henan Provincial People’s Hospital, People’s Hospital of Zhengzhou University, Zhengzhou, China

**Keywords:** insomnia disorder, real-time fMRI, neurofeedback, degree centrality, functional connectivity

## Abstract

**Background:**

Chronic insomnia disorder (CID) is considered a major public health problem worldwide. Therefore, innovative and effective technical methods for studying the pathogenesis and clinical comprehensive treatment of CID are urgently needed.

**Methods:**

Real-time fMRI neurofeedback (rtfMRI-NF), a new intervention, was used to train 28 patients with CID to regulate their amygdala activity for three sessions in 6 weeks. Resting-state fMRI data were collected before and after training. Then, voxel-based degree centrality (DC) method was used to explore the effect of rtfMRI-NF training. For regions with altered DC, we determined the specific connections to other regions that most strongly contributed to altered functional networks based on DC. Furthermore, the relationships between the DC value of the altered regions and changes in clinical variables were determined.

**Results:**

Patients with CID showed increased DC in the right postcentral gyrus, Rolandic operculum, insula, and superior parietal gyrus and decreased DC in the right supramarginal gyrus, inferior parietal gyrus, angular gyrus, middle occipital gyrus, and middle temporal gyrus. Seed-based functional connectivity analyses based on the altered DC regions showed more details about the altered functional networks. Clinical scores in Pittsburgh sleep quality index, insomnia severity index (ISI), Beck depression inventory, and Hamilton anxiety scale decreased. Furthermore, a remarkable positive correlation was found between the changed ISI score and DC values of the right insula.

**Conclusions:**

This study confirmed that amygdala-based rtfMRI-NF training altered the intrinsic functional hubs, which reshaped the abnormal functional connections caused by insomnia and improved the sleep of patients with CID. These findings contribute to our understanding of the neurobiological mechanism of rtfMRI-NF in insomnia treatment. However, additional double-blinded controlled clinical trials with larger sample sizes need to be conducted to confirm the effect of rtfMRI-NF from this initial study.

## Introduction

Chronic insomnia disorder (CID) is considered a major public health problem worldwide and is characterized by difficulty in falling asleep at bedtime, frequent awakening in the middle of the night, and waking up early in the morning (Buysse et al., 2017). These symptoms, which persist over at least 3 months, reduce the quality of daily life, affect work efficiency, and cause mental symptoms, such as depression and anxiety, which might become life threatening (Spiegelhalde et al., 2015; Buysse et al., 2017). However, the pathogenesis of insomnia is still unclear, causing challenges to its treatment (Kay and Buysse, 2017). Therefore, innovative therapies and pathogenesis of CID need to be studied.

The current treatment for CID includes drug and non-drug therapies (Buysse et al., 2017; Sateia et al., 2017). Long-term clinical drug treatment has limitations, causing a series of side effects and drug dependence (Buysse et al., 2017; Zhao et al., 2020). The “American College of Physicians Guidelines for the Clinical Diagnosis and Treatment of Insomnia in Adults” and the “Guidelines for the Diagnosis and Treatment of Insomnia in Adults in China” recommend non-pharmacological treatments as first-line treatments for CID (Qaseem et al., 2016; Buysse et al., 2017). The main clinical non-drug adjuvant treatments for insomnia are cognitive behavioral therapy (CBT) and neurofeedback (Buysse et al., 2017; Zhao et al., 2020). CBT improves sleep through interviews, psychological counseling, and changes in sleep habits. However, this method has a certain degree of blindness. Moreover, it cannot observe changes in brain activity in real time and clarify changes in brain neurobiology and its effective neurobiological mechanisms (Zhao et al., 2020). The neurofeedback method based on electroencephalogram (EEG) signals can overcome the shortcomings of CBT, but the spatial resolution is low; it cannot record EEG signals from the deep brain areas (Weiskopf, 2012; Samantha et al., 2020; Zhao et al., 2020). Hence, innovative and effective technical methods for studying the pathogenesis and clinical comprehensive treatment of CID need to be developed urgently.

Real-time fMRI neurofeedback (rtfMRI-NF) is a new intervention with great application value that trains subjects to adjust brain neural activity autonomously to improve cognition or cure diseases (Weiskopf, 2012; Watanabe et al., 2017; Samantha et al., 2020). In comparison with other neurofeedback techniques, such as EEG and non-invasive physical stimulation techniques (e.g., transcranial magnetic stimulation, TMS), rtfMRI-NF has higher spatial resolution and localization accuracy and better access to deep relevant brain structures (Weiskopf, 2012; Watanabe et al., 2017; Samantha et al., 2020). This technology enables subjects to regulate brain activity and improve the clinical symptoms of major depressive disorder (MDD), anxiety, schizophrenia, and other diseases (Morgenroth et al., 2020; Tursic et al., 2020; Tsuchiyagaito et al., 2021). rtfMRI-NF may be an efficient method for the treatment of patients with CID (Spiegelhalde et al., 2015). However, this technology has not been applied in CID treatment.

Dysfunctional emotional responses might mediate the interaction between cognitive and autonomic hyperarousal, thus maintaining insomnia (Baglioni et al., 2010). Moreover, dysfunctions in sleep–wake-regulating neural circuitries can reinforce emotional disturbances (Baglioni et al., 2010). The use of fMRI technology in exploring the deep neural functional changes in CID has revealed multiple local and overall dysfunctions in the brain, which are concentrated in the amygdala and other emotion- and cognition-related brain areas (Huang et al., 2012; Baglioni et al., 2014; Spiegelhalde et al., 2015). Baglioni et al. (2014) found that CID is associated with increased amygdala responsiveness to negative stimuli, and insomnia treatment may benefit from strategies that modulate its association with emotion. By using resting-state fMRI, Huang et al. (2012) found that the amygdala has decreased functional connectivity (FC) with the insula, striatum, and thalamus and increased FC with the premotor and sensorimotor cortex. The amygdala is located in the center of the limbic system, and it plays a key role in the generation and expression of emotions and the perception of negative emotions; thus, the amygdala may be involved in the pathogenesis of CID (Pessoa, 2010). Many researchers have successfully used rtfMRI-NF training to help patients regulate the activity of the left amygdala through positive autobiographical memory to change brain function and clinical symptoms (Watanabe et al., 2017; Samantha et al., 2020; Tursic et al., 2020). Therefore, the selection of amygdala activity as the target of rtfMRI-NF regulation may improve sleep in CID and provide a new breakthrough point for studying neural mechanism.

Recently, resting-state fMRI has been increasingly used to address changes in brain FC following effective treatments (Dichter et al., 2015). Resting-state FC is a highly effective and sensitive method for mapping complex neural circuits that may reflect the underlying neurobiological mechanism (Wang et al., 2012; Fasiello et al., 2021). Altered FC could be calculated by comparing resting-state FC during different stages (e.g., before and after training) and may indicate the effect of rtfMRI-NF training. Voxel-wise degree centrality (DC) is a data-driven method used to measure the FC number of a given voxel with all other voxels within the entire brain, thus enabling the identification of FC hubs in the human brain (Yan et al., 2018, 2020). Unlike seed-based or independent component analysis (ICA) approaches, voxel-wise DC was developed to reveal regions with consistent global connections even when the individual connections vary across regions for different subjects or patients (Yan et al., 2018, 2020). Voxel-based DC has high sensitivity, specificity, and test–retest reliability and has been widely used to investigate diseases such as CID, MDD, Alzheimer’s disease, and Parkinson’s disease (Zuo and Xing, 2014; Yan et al., 2018, 2020). Thus, voxel-wise DC analysis could be used to gain insight into the neural mechanisms underlying rtfMRI-NF training.

Taken together, we hypothesized that amygdala-based rtfMRI-NF training could change DC and improve the sleep of patients with CID. rtfMRI-NF was used to train patients with CID to regulate their amygdala activity for three sessions in 3 weeks to test our hypothesis. Resting-state fMRI data were collected before and after training. Then, voxel-based DC method was used to explore the effect of training. For regions with altered DC, we determined the specific connections to other regions that most strongly contributed to altered functional hubs based on DC. We also investigated the relationships between the DC value of altered regions and changes in clinical variables.

## Materials and Methods

### Participants

This study was approved by the Ethics Committee of Henan Provincial People’s Hospital. All patients with CID were outpatients from the neurology department of the hospital or recruited *via* advertising. These patients were recruited from January 2018 to December 2021. All participants provided written informed consent to participate in the study and received equal financial compensation. The subjects underwent complete physical and neurological examination, standard laboratory tests, and certain psychological assessments, such as Pittsburgh sleep quality index (PSQI), insomnia severity index (ISI), Hamilton depression scale (HAMD), Beck depression inventory (BDI), and Hamilton anxiety scale (HAMA). Moreover, the participants should meet the criteria for CID in the fifth edition of Diagnostic and Statistical Manual of Mental Disorders and should not have taken medication that would influence brain function 2 weeks before the experiment. The inclusion criteria are as follows: (1) duration of insomnia symptoms, such as fatigue, testiness, or cognitive decline, of no less than 3 months, (2) PSQI score ≥ 8, (3) no neurological or psychiatric disorders, such as stroke, (4) no other sleep disorders such as sleep-related movement disorders, hypersomnia, or parasomnia, (5) right-hand dominance and native Chinese speaker, (6) 18–70 years old, (7) no medication or substance abuse, such as excessive intake of caffeine, nicotine, or alcohol, and (8) no abnormal signal found by T2-weighted dark-fluid and T1-weighted MR images. Furthermore, overnight polysomnography (PSG) was performed using an ambulatory recording system (Compumedics Siesta, Australia) to exclude participants with occult sleep disorders other than insomnia. PSG characteristics, including total sleep time, sleep efficiency, sleep onset latency, and the number of awakenings, were collected. Finally, 33 subjects participated and completed all the procedures of our experiment. However, four subjects were excluded because of large head motion during the resting-state scan. One subject dropped out because of a pause in training.

### Procedure

**FIGURE 1:**
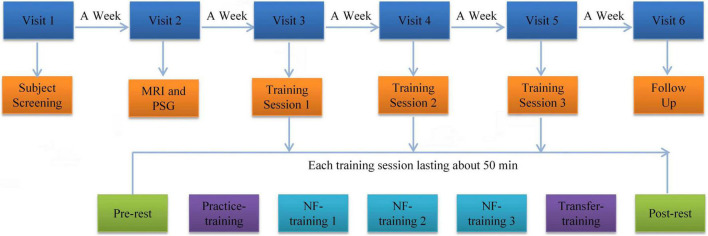
Procedure of rtfMRI-NF training experiment. The experimental protocol consisted of six visits lasting for approximately 35 days. Each rtfMRI-NF training session included seven runs lasting for approximately 50 min. The pre- and post-rest runs were carried out for 7 min. The five remaining runs were carried out for 6 min and 50 s. rtfMRI-NF, real-time fMRI neurofeedback.

The experimental procedure is shown in Figure 1. Participants were required to visit the hospital six times to complete the experiment. During visit 1, we collected the general demographic characteristics of the participants. The participants also completed PSQI, ISI, HAMD, BDI, and HAMA. During visit 2, all subjects underwent MRI scans and were familiar with the MRI scanning environment. Routine axial T2-weighted dark-fluid and T1-weighted MR images were acquired to exclude brain structure abnormality. High-resolution T1-weighted structural images were acquired to register the template of the amygdala from the standard space to the subject space for real-time processing data during training. Afterward, overnight PSG was performed. During visit 3, 4, and 5, the participants completed the same clinical and self-report measures as visit 1 and their three rtfMRI-NF training sessions. The following section details the rtfMRI-NF training process. During visit 6, the participants completed resting-state fMRI scan, overnight PSG, the same clinical, and self-report measures similar to visit 3. An interval of approximately 1 week from the previous visit was set. Throughout the training period, the subjects were required not to take drugs that help sleep. The subjects were informed that they can withdraw from the training at any time.

### Real-Time fMRI Neurofeedback Training Paradigm

Before rtfMRI-NF training, we asked the subjects to write down three or more specific autobiographical memories about themselves and explained the specific tasks under the experimental stimulation. They received information on brain activity signals from the left amygdala region, as defined from the Talairach space with a radius of 7 mm and the coordinates (−21, −5, −16) (Young et al., 2017). Amygdala activity was displayed as temperature bars and updated once per retention time (TR, 2 s). Real-time online data-processing was performed using OpenNFT system (Koush et al., 2017). The detailed steps and parameters are provided in the paper published by Koush et al. (2017). Each training session has seven runs, including pre-rest, practice training, three NF training, transfer training, and post-rest, which lasted for approximately 50 min. Pre-rest and post-rest were carried out for 7 min. All the five other runs were carried out for 6 min and 50 s. Resting-state fMRI data were collected in the pre-rest and post-rest runs. During the resting-state fMRI data acquisition, all subjects were instructed to fix their vision on the green cross, keep awake, and think of nothing in particular. The practice and transfer training runs shared the same paradigms with NF training but without a feedback signal. The practice training run was designed to familiarize the subjects with the training procedures. The transfer training run was designed to test whether the subjects had mastered the regulation strategy. Each NF training run consisted of alternating 30 s of rest and 30 s of happy blocks with seven rest blocks and six happy blocks. During rest blocks, the subjects were asked to stare at the green cross on the screen to calm their mind. During happy blocks, the subjects were instructed to increase the height of the thermometer on the screen by recalling a positive autobiographical memory.

### Data Acquisition

All fMRI data were acquired using a MAGNETOM Prisma 3T MR scanner (Siemens Healthcare, Erlangen, Germany) with a 64-channel head–neck coil at the Medical Imaging Center of our hospital. Foam pads were used to minimize the subjects’ head motions and diminish scanner noise. Medical tape was attached to their foreheads for them to feel their head movements and prevent out-of-range movements. Routine axial T2-weighted dark-fluid and T1-weighted MR images were acquired. fMRI data were acquired using an echo-planar imaging sequence with the following parameters: TR = 2,000 ms, echo time (TE) = 30 ms, field of vision (FOV) = 224 mm × 224 mm, matrix size = 112 × 112, slices = 27, slice thickness = 4 mm, flip angle = 90°. In total, 210 volumes lasting for 420 s were collected. High-resolution T1-weighted structural images were acquired with the following parameters: TR = 2,300 ms, TE = 2.27 ms, FOV = 250 mm × 250 mm, matrix size = 256 × 256, slices = 192, slice thickness = 1 mm, flip angle = 8°.

### Data Processing

The resting-state fMRI data collected in visit 6 and the pre-rest run of visit 3 were used for analysis in the present study. Image pre-processing was performed using Data Processing and Analysis of Brain Imaging (DPABI, version 4.3) toolbox (Yan et al., 2016). First, the removal of the first 10 volumes, slice timing, and head motion correction were done by preprocessing functional data. Data with a maximum displacement in head rotation of larger than 2° or any directions larger than 2 mm were excluded from further analysis. For the precise spatial normalization of the fMRI data, individual high-resolution T1-anatomic images were registered to the mean fMRI data, and the resulting aligned T1-weighted images were segmented and transformed into standard Montreal Neurological Institute space by using the DARTEL toolbox. Furthermore, white matter and cerebrospinal fluid signals and 24 head realignment parameters were regressed out as covariates. Subsequently, the regressed functional images were normalized to the group template by using the transfer parameter estimated by DARTEL segmentation and resampled to 3 × 3 × 3 mm^3^ voxels. Finally, linear trend and temporal band-pass filtering (0.01–0.1 Hz) was applied.

### Degree Centrality Calculation

Degree centrality maps were generated *via* voxel-based whole-brain correlation analysis on the preprocessed resting-state fMRI data as previously described (Yan et al., 2018, 2020). The Pearson’s correlation coefficient (*r*) between each pair of brain gray matter voxels was computed. A binary undirected correlation matrix was obtained by thresholding each correlation at *r* > 0.25 to remove the weak correlations caused by noises. DC maps were also calculated for the thresholds at *r* = 0.15, 0.20, 0.30, 0.35 to assess the robustness of the chosen threshold (Zuo and Xing, 2014; Yan et al., 2018, 2020). Only positive correlations were considered in the DC calculations. Then, the DC maps of individuals were converted into z-score maps *via* Fisher-Z transformation. Finally, the DC maps were spatially smoothed with a smooth kernel of 6 mm. A group template of 90% was generated for fMRI processing and statistics. The weighted version of DC was also computed. The whole-brain DC differences between post- and pre-training conditions were compared using paired *t*-test to assess the effect of rtfMRI-NF on the brain of patients with CID. Based on previous studies that used the DC method on CID, Gaussian random field (GRF) theory correction procedure was used for multiple comparisons (Huang et al., 2017; Liu et al., 2018; Yan et al., 2018).

### Seed-Based Functional Connectivity Calculation

Seed-based interregional correlation analysis was performed using the DPABI software package to explore more details about resting-state FC alterations. The seed regions were defined based on altered DC regions (post-training vs. pre-training) by drawing a 6 mm-radius sphere region of interest (ROI) around the activated center of mass coordinates (Gao et al., 2021). By using the DPABI toolbox, we calculated the FC maps by correlating the mean time series of all voxels within the ROI to the time courses of all brain voxels in the gray matter mask. Afterward, Fisher-Z transform analysis was applied to the FC maps to obtain an approximately normal distribution. Finally, a spatially smooth step was added to the z-score FC maps by using a 6 mm kernel. Paired *t*-test was used to compare the differences of FC maps between post- and pre-training conditions.

### Statistical Analysis

#### Demographic and Clinical Data Analysis

Statistical analysis of demographic and clinical data was performed using SPSS version 22.0 (Chicago, IL, United States). The threshold for statistical significance was set at *p* < 0.05, and all hypothesis tests were two-tailed. The distribution of clinical data and DC values were tested using Kolmogorov–Smirnov method. Continuous variables with normal distribution were analyzed using independent paired *t*-test and expressed as mean ± standard deviation. Otherwise, Wilcoxon signed-rank test was used to analyze the data with non-normal distribution, and the results are expressed as median and interquartile range.

#### Brain–Behavior Correlation Analysis

The averaged DC values of altered brain regions in post-training were extracted to calculate the Partial (normally distributed data) or Spearman (non-normally distributed data) correlation between DC values and changes in clinical scores and PSG indexes, including PSQI, ISI, HAMD, HAMA, BDI, total sleep time, sleep efficiency, sleep onset latency, and number of awakenings, with age, gender, and education as covariates. Statistical significance was considered at *p* < 0.05.

## Results

### Demographic and Clinical Data

Finally, the data of 28 patients with CID were analyzed in this paper (7 males; age: 45.7 ± 13.2 years; education: 13.1 ± 3.3 years). The clinical characteristics (PSQI, ISI, HAMD, HAMA, BDI, and total sleep time) and DC values of the altered regions were normally distributed. Sleep efficiency, sleep onset latency, and the number of awakenings had a non-normal distribution. In comparison with pre-training, patients with CID showed significant differences in PSQI, ISI, BDI, and HAMA in post-training (*p* < 0.05; Table 1). However, HAMD, total sleep time, sleep efficiency, sleep onset latency, and the number of awakenings showed no remarkable differences.

**TABLE 1:** Clinical characteristics of pre-training and post-training.

Variables	Pre-training	Post-training	T/Z value	*p* value
PSQI (score)	13.86 ± 3.35	11.25 ± 3.31	4.87	0.000
ISI (score)	17.82 ± 4.83	14.18 ± 6.14	3.463	0.002
HAMD (score)	19.11 ± 8.01	16.86 ± 7.47	1.759	0.090
BDI (score)	19.29 ± 10.70	15.89 ± 9.25	2.878	0.008
HAMA (score)	19.82 ± 9.45	16 ± 12.15	2.653	0.013
Total sleep time (min)	380.11 ± 101.70	404.27 ± 81.22	1.176	0.250
Sleep efficiency (%)	78.75 (66.25–84.78)	78.6 (71.33–89.98)	–0.615	0.539
Sleep onset latency (min)	20.5 (7.25–58.13)	12 (6.63–27.13)	–1.946	0.052
Number of awakenings	20.5 (10.25–26.75)	15 (8–26.5)	–1.488	0.137

*Normal distribution data is presented as mean ± SD and p values were obtained by two-tailed paired t-test. Nor-normal distribution data is presented as median and inter-quartile range and p values were obtained by Wilcoxon signed-rank test. PSQI, Pittsburgh sleep quality index; ISI, insomnia severity index; HAMD, Hamilton Depression Rating Scale; BDI, Beck depression inventory; HAMA, Hamilton Anxiety Rating Scale.*

### Degree Centrality Analysis

**FIGURE 2:**
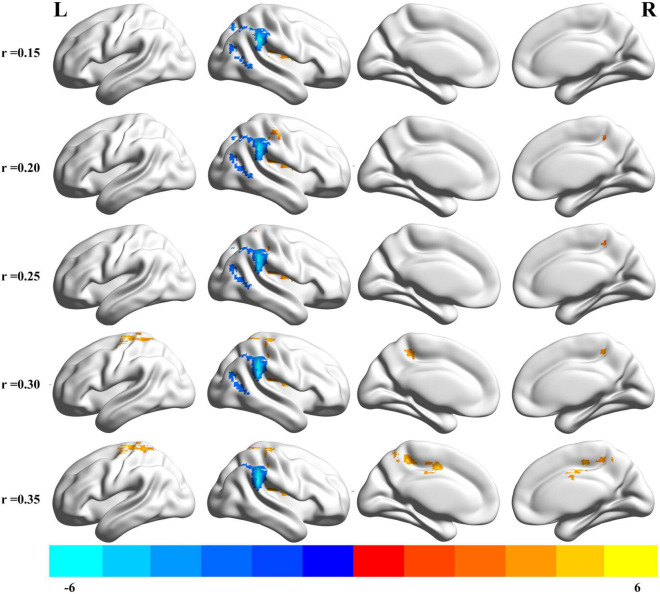
Brain areas that exhibited altered binary DC induced by real-time fMRI neurofeedback training using different cut off thresholds (*r* = 0.15, 0.20, 0.25, 0.30, 0.35). Results were set at voxel-level *p* < 0.01, cluster-level *p* < 0.05, and *t* = 2.77 (Gaussian random field corrected). Warm colors indicate regions in which DC remarkably increased, whereas cool colors indicate regions in which DC remarkably decreased. The color bar indicates the *t* value. DC, degree centrality; L, left; R, right.

The results of altered binary DC maps are shown in Figure 2. Detailed information on activation centers are provided in Table 2 and Supplementary Table 1. All results were set at voxel-level (*p* < 0.01), cluster-level (*p* < 0.05), and *t* = 2.77 (GRF-corrected). The results clearly showed a highly similar altered binary DC in several thresholds at *r* = 0.15, 0.20, 0.25, 0.30, 0.35. Besides, the weighted version of DC, which assures the robustness of the findings with nearly identical results, is shown in Supplementary Figure 1. The present study reports the results of DC at the correlation threshold of 0.25 as previously described (Yan et al., 2018, 2020). After rtfMRI-NF training, DC increased in the right postcentral gyrus (PoCG), Rolandic operculum (ROL), insula, and superior parietal gyrus (SPG) and decreased in the right supramarginal gyrus (SMG), inferior parietal gyrus (IPG), angular gyrus (ANG), middle occipital gyrus (MOG), and middle temporal gyrus (MTG).

**TABLE 2:** Brain regions exhibited altered DC at *r* = 0.25 (post-training vs. pre-training).

Brain regions	Whole cluster size	Cluster size	MNI coordinates			*t* score
			x	y	z	
R Postcentral gyrus	405	76	39	−30	42	3.961
R Rolandic operculum		71	48	−21	21	5.865
R Insula		56	33	−6	12	5.388
R Superior parietal gyrus		20	24	−51	60	3.821
R Supramarginal gyrus	524	157	60	−42	36	–7.105
R Inferior parietal gyrus		99	51	−42	45	–4.831
R Angular gyrus		86	33	−54	42	–4.883
R Middle occipital gyrus		60	33	−75	30	–3.688
R Middle temporal gyrus		46	48	−69	21	–3.527

*Results were set at voxel-level: p < 0.01, cluster-level: p < 0.05, t = 2.77 (Gaussian random field corrected). DC, degree centrality; R, right; MNI, Montreal Neurological Institute.*

### Seed-Based Functional Connectivity Analysis

**FIGURE 3:**
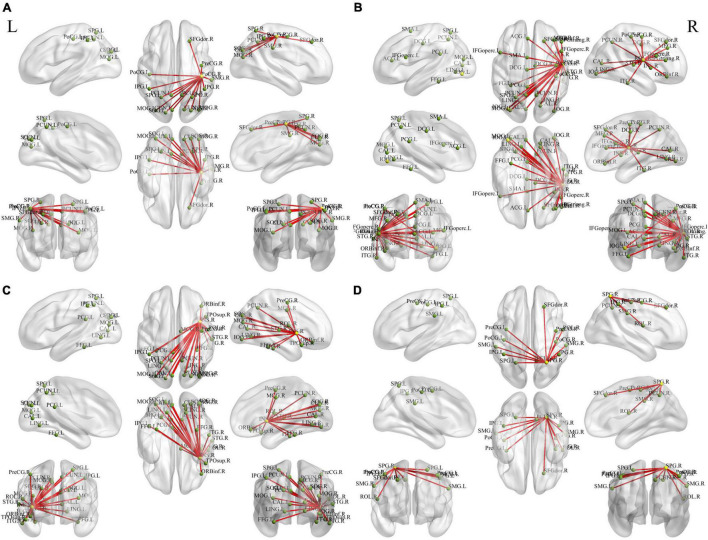
Holistic view of the differences in seed-based (increased DC regions) FC between pre- and post-training in patients with chronic insomnia disorder. Results were set at voxel level *p* < 0.01, cluster-level *p* < 0.05, and *t* = 2.77 (Gaussian random field corrected). The seed regions are represented by yellow nodes, including the right postcentral gyrus **(A)**, Rolandic operculum **(B)**, insula **(C)**, and superior parietal gyrus **(D)**. The red lines represent increased FC strength. The abbreviations for brain regions are listed in Supplementary Table 2. DC, degree centrality; FC, functional connectivity; L, left; R, right.

**FIGURE 4:**
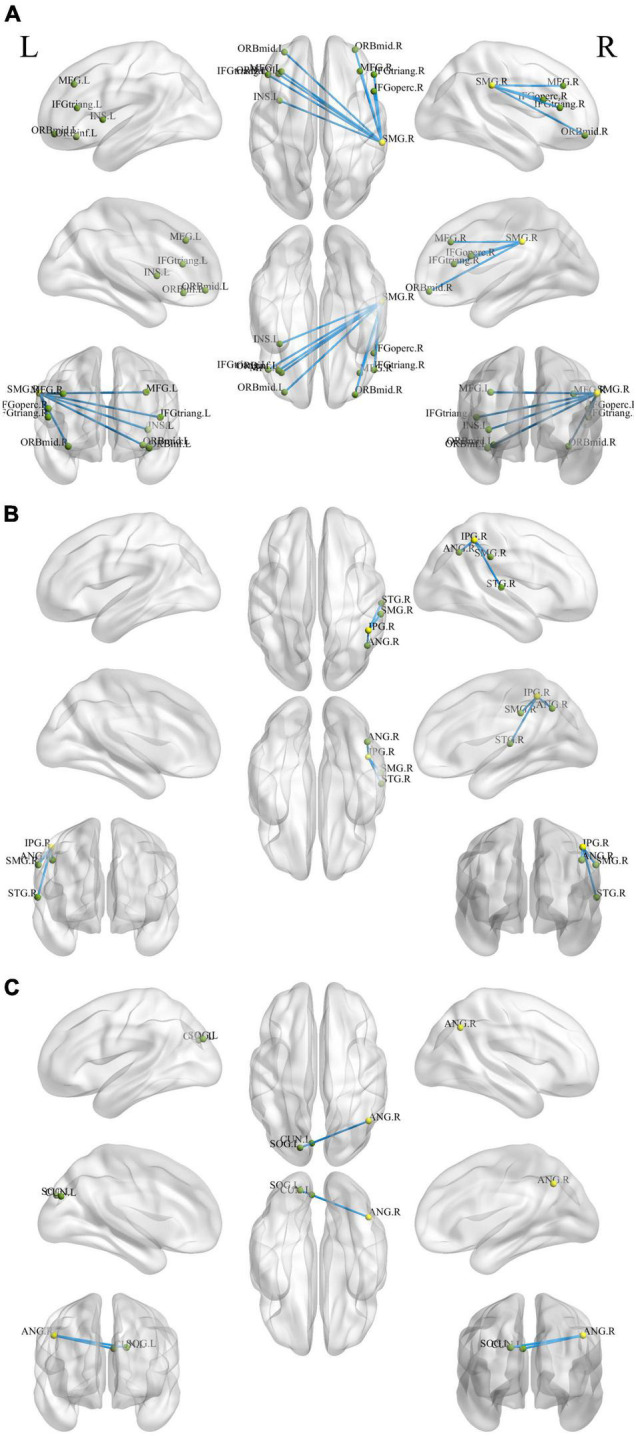
Holistic view of the differences in seed-based (decreased DC regions) FC between pre- and post-training in patients with chronic insomnia disorder. Results were set at voxel level *p* < 0.01, cluster-level *p* < 0.05, and *t* = 2.77 (Gaussian random field corrected). The seed regions are represented by yellow nodes, including the right supramarginal gyrus **(A)**, inferior parietal gyrus **(B)**, and angular gyrus **(C)**. The blue lines represent decreased FC strength. The abbreviations for brain regions are listed in Supplementary Table 3. DC, degree centrality; FC, functional connectivity; L, left; R, right.

We examined the FC of altered DC regions with whole-brain regions to backtrack the connectivity patterns. Results were set at voxel-level (*p* < 0.01), cluster-level (*p* < 0.05), and *t* = 2.77 (GRF-corrected). The detailed information of the activated brain regions is specified in Figures 3, 4 and Supplementary Tables 2, 3. Our results indicate increased or decreased FC with regions of altered DC across the whole brain. Increased FC was observed within the prefrontal cortex, frontal cortex, parietal cortex, occipital cortex, temporal cortex, and subcortex. Decreased FC was observed within the prefrontal cortex, parietal cortex, occipital cortex, temporal cortex, and subcortex. However, no cluster survived at voxel-level *p* < 0.01 in the seeds of MOG and MTG.

### Brain–Behavior Correlation Analysis

**FIGURE 5:**
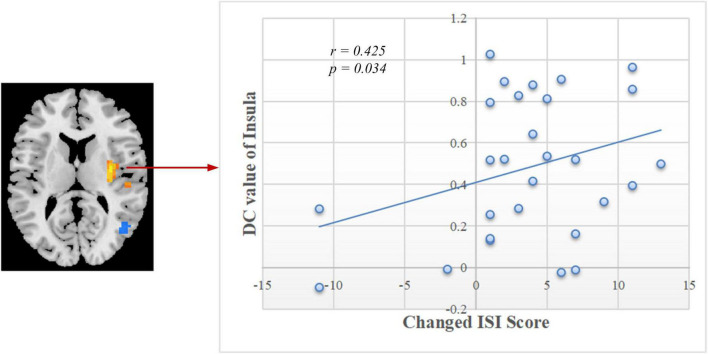
Correlation between the changed ISI scores and DC values of the right insula in post-training. ISI, Insomnia Severity Index; DC, degree centrality.

As shown in Figure 5, significant positive correlation was found between the changed ISI score (post-training minus pre-training) and DC values of the right insula after rtfMRI-NF training (*r* = 0.425, *p* = 0.034). The other changed clinical scores and indexes of PSG had no remarkable correlations with the DC values of altered brain regions.

## Discussion

By using resting-state fMRI technology combined with voxel-vise DC approach, we explored whether the functional hubs in patients with CID could be regulated by rtfMRI-NF training with improved clinical symptoms. The results validated our prior hypothesis that patients with CID showed altered DC and decreased clinical scores after rtfMRI-NF training. DC increased in the right PoCG, ROL, insula, and SPG and decreased in the right SMG, IPG, ANG, MOG, and MTG. The seed-based FC analyses based on the altered DC regions showed more details about the altered functional networks. Clinical scores, including PSQI, ISI, BDI, and HAMA scores, decreased. Furthermore, a remarkable positive correlation was found between the changed ISI score and DC values of the right insula after rtfMRI-NF training. These results support our hypothesis that rtfMRI-NF training could change brain DC and improve the sleep of patients with CID.

The insula of the salience network plays key roles in saliency detection, decision-making, motor/sensory processes, emotion and attention regulation, and cognition (Uddin, 2015). Patients with CID exhibit insula abnormalities in task and rest (Martin et al., 2015; Liu et al., 2016; Lu et al., 2017). During an immediate and impulsive monetary decision task, patients with CID show smaller activations in the bilateral insula than HCs (Martin et al., 2015). Liu et al. (2016) found a decreased fractional amplitude of low-frequency fluctuation (ALFF) in the left anterior insula and bilateral posterior insula. In comparison with the non-early improvement group, the early improvement group following treatment with antidepressants had stronger FC between the right anterior insula and left dorsolateral prefrontal cortex (Yuan et al., 2020). Therefore, the resting-state FC between the insula and other regions may represent an early symptom improvement in self-perceptual anxiety and insomnia. By using the insula as the target of rtfMRI-NF training, Yao et al. (2016) discovered an increase in the FC of the insula with the superior temporal gyrus, IPG, posterior cingulate cortex, insula, and cuneus after training. The enhanced FC network in our study is consistent with the above study (Figure 3C and Supplementary Table 2). By using the same method of voxel-wise DC, Huang et al. (2017) and Liu et al. (2018) both found decreased FC in the bilateral insula of patients with CID and a positive linear correlation between the strength of FC pairs between the right and left insula and the HAMA score. Notably, the DC of the right insula decreased, and a significant positive correlation (*r* = 0.425, *p* = 0.034, Figure 5) was found between the changed ISI score and DC values of the right insula after rtfMRI-NF training. In summary, rtfMRI-NF training may enhance the FC of the insula in patients with CID within these networks, and this phenomenon is associated with a broad range of emotional processing and emotion regulation ability to improve sleep. Moreover, the insula may be a target for rtfMRI-NF training. However, more studies are needed to confirm these findings.

In the present study, we discovered that patients with CID showed increased DC in the parietal cortex, including PoCG and SPG. The PoCG is the main receptive region for external stimuli as the location of the primary somatosensory cortex (Joo et al., 2013). Gray matter volume (GMV) in the right PoCG is reduced in patients with CID (Joo et al., 2013; Chou et al., 2021). Moreover, sleep latency and quality are negatively correlated with GMV in the right PoCG (Joo et al., 2013; Chou et al., 2021). Relative to HCs, regions with decreased integrity of structural covariance were observed in the bilateral PoCG (Chou et al., 2021). Therefore, the PoCG structure network of the patient with CID was disrupted. By using the bilateral PoCG as seed, Dai et al. (2020) reported decreased FC with cuneus (positively correlated with CID duration) and with superior frontal gyrus (SFG). A recent meta-analysis also found reduced frontal–parietal activation following sleep deprivation (Ma et al., 2015). Based on seed-based FC analysis, our study found that the right PoCG showed increased FC with the cuneus and dorsolateral SFG (Figure 3A and Supplementary Table 2). Therefore, rtfMRI-NF training could reshape the disrupted FC of PoCG. Thus, the increased DC of the PoCG suggests that rtfMRI-NF training may enhance the patient’s ability to process external information.

The SPG is critically important for the manipulation of information in spatial working memory (Koenigs et al., 2009). The SPG of patients with CID showed lower activation during a spatial memory task (Li Y. et al., 2016). Based on seed-based FC analysis, patients with CID had weaker connectivity between the right SFG and bilateral SPG (Li et al., 2014). Li et al. (2018) reported reduced FC in the regions of the right fronto-parietal network, including SFG and SPG, in patients with CID compared with HC. Patients with CID have deficits in working memory tasks and several attentional processes (Koenigs et al., 2009; Li et al., 2014, 2018; Li Y. et al., 2016). After rtfMRI-NF training, we found that the SPG had enhanced interaction with regions in the default mode network (DMN), including the dorsolateral SFG, IPG, and precuneus (Figure 3D and Supplementary Table 2). Kay et al. (2016) found that the SPG with abnormal metabolism might be a meaningful target for cognitive training, mindfulness meditation, or repetitive TMS. Therefore, the increased DC of the SPG might enhance the working memory and attention process capacity of patients with CID.

The ROL plays a recognized role in various neurologic and psychiatric conditions (Mălîia et al., 2018; Sutoko et al., 2020). Its complex functions include sensory, motor, autonomic, cognitive, and emotion processing. These functions are implemented by highly specialized neuronal populations and their widespread connections (Mălîia et al., 2018). Sutoko et al. (2020) found that the high severity level in other psychological domains (e.g., apathy, depression, and anxiety) is associated with the high lesion degree at the right ROL. Janse et al. (2019) reported that compared with neutral images, the ROL has reduced neural response to emotional images in alcohol-dependent patients versus HCs. In comparison with HCs, Huang et al. (2017) found decreased FC between left insula and left ROL in patients with CID. Moreover, simultaneous enhancement and reduction of connections were observed with the right ROL (Fasiello et al., 2021). In the present study, increased FC between right ROL and right insula was found after rtfMRI-NF training (Figure 3B and Supplementary Table 2). Hence, rtfMRI-NF training may enhance the CID emotion processing ability by enhancing the ROL’s connections with other related brain regions.

The MTG, IPG, ANG, and SMG are important hubs of DMN (Raichle et al., 2001; Fox and Raichle, 2007; Nie et al., 2015) that showed decreased DC after rtfMRI-NF training. The DMN plays a central role in the modulation of consciousness and is associated with self-referential mental activity, emotional and episodic memory processing, or mind wandering when individuals are not focused on the external environment (Raichle et al., 2001; Nie et al., 2015). CID may be conceptualized as a disorder associated with the overactivity of certain brain areas of the DMN (Buysse et al., 2011; Marques et al., 2015). The widespread hyperarousal of several systems (e.g., cognitive, physiological, emotional) occurs during insomnia, thus preventing relaxation (Marques et al., 2015). The MTG is related to declarative memory, information retrieval, and cognitive processes (Squire et al., 2004; Wang et al., 2017). Patients with CID showed high regional homogeneity and ALFF values in the MTG (Dai et al., 2014; Li C. et al., 2016; Zhou et al., 2017). During an eye-closed resting-state condition, a high-density EEG study reported that patients with CID have higher beta activity than HCs in the right temporal lobe (Colombo et al., 2016). In comparison with HCs, patients with CID showed an increased response in the DMN (including right MTG) upon exposure to self-related words (Marques et al., 2018). Zhou et al. (2020) discovered increased short DC in the MTG compared with HCs. By using the ICA method, Marques et al. (2017) found that patients with CID exhibited increased activation in the right MTG and IPG compared with HCs. The IPG is a multimodal complex that receives somatosensory, visual, and auditory inputs and consists of the ANG and SMG (Leichnetz, 2011; Lee et al., 2018). Zhou et al. (2017) discovered that the ALFF of the right IPG increased, and this phenomenon is related to lower sleep quality and higher anxiety.

The ANG belongs to the posterior heteromodal association cortex and is involved in various cognitive functions, including attention, memory retrieval, conflict resolution, and theory of mind (Wei et al., 2019). Wei et al. (2019) reported that the right ANG is a hub of enhanced structural connectivity in CID. Hyperconnectivity within the identified subnetwork may contribute to increased reactivity to stimuli and may signify vulnerability to CID (Wei et al., 2019). The SMG and ANG share a similar role in somatosensory functions (Bjoertomt et al., 2009). Sleep diary-assessed sleep efficiency is inversely related to relative glucose metabolism in the right SMG (Kay et al., 2016). In comparison with the HCs, the CID group showed increased locus coeruleus noradrenergic FC in the left SMG and left MOG (Gong et al., 2021). The MOG is involved in visual and spatial information processing (Renier et al., 2010). These results support the hyperarousal hypothesis, in which patients with CID often show hyperperfusion in the sensory perception of tactile, visual, and auditory stimuli (Kay and Buysse, 2017; Gong et al., 2021). After rtfMRI-NF training, the FC of the SMG decreased with brain regions located in the prefrontal and subcortical regions, including the middle frontal gyrus, inferior frontal gyrus, and insula (Figure 4C and Supplementary Table 3). The FC of IPG with SMG and ANG also decreased (Figure 4B and Supplementary Table 3). The reduced DC of the right IFG, MTG, ANG, and SMG indicate a decreased interaction with other regions across the whole brain, and this condition may inhibit the processing of sensory information to avoid excessive activation, indicating the potential neurobiological mechanism in which rtfMRI-NF training improves the sleep of patients with CID.

After the intervention by amygdala-based rtfMRI-NF, the BDI, HAMA, ISI, and PSQI decreased, suggesting the improvement of mood state and sleep. During training, the subjects tried to increase the activity of amygdala by recalling positive autobiographical memory, which may enhance the affective or attentional significance of these memories. The amygdala is a critical region of the neural circuitry for emotion and is part of the salience network (Pessoa, 2010; Young et al., 2017). After training, we discovered increased DC region (insula) in the salience network, and altered DC regions outside it. These results indicated that positive autobiographical memory may promote the activity of amygdala and emotion regulation related brain circuits. The synergy between amygdala activity and positive autobiographical memory may drive the clinical improvements of mood state and sleep. The CID has a close relationship with depression and anxiety (Alvaro et al., 2013). Therefore, in the future treatment of CID, the patient’s anxiety and depression should be considered at the same time.

## Conclusion

In summary, our findings support our hypothesis that amygdala-based rtfMRI-NF training altered the intrinsic functional hubs and improved the sleep of patients with CID. Based on voxel-wise and seed-based FC, we discovered that rtfMRI-NF training reshaped the abnormal FC caused by insomnia. These findings improved our understanding of the neurobiological mechanism of rtfMRI-NF in the treatment of insomnia. However, additional double-blinded controlled clinical trials with larger sample sizes are necessary to confirm the effect of rtfMRI-NF from this initial study.

## Limitations and Strengths

Several limitations should be considered in our study. First, a sham control group was not used, thus possibly decreasing the credibility of the conclusion. Patients with CID participating in experiment hope to improve sleep, thereby reducing the suffering of insomnia. Their compliance is greatly reduced; if they find the effect as not significant, they may realize that they are in the sham feedback group. Therefore, only an amygdala-based feedback group was included to ensure the training effect of patients with CID in this initial study. Accordingly, we intend to add three sham feedback sessions before three real feedback sessions in the future study. Second, the sample size of this study was small. Larger sample sizes are necessary to confirm the credibility of our results in this initial study. Third, this paper used a relatively loose threshold. Considering the lack of HC, the threshold was set at voxel-level *p* < 0.01 for comparing our results with previous studies (Huang et al., 2017; Liu et al., 2018; Yan et al., 2018), which may result in a high risk of false positives. However, when the threshold was set at voxel-level *p* < 0.001 (cluster-level *p* < 0.05, *t* = 3.29, GRF-corrected), the main altered brain regions remained, including ROL, insula, SMG, and IPG, except for PoCG, SPG, ANG, MOG, and MTG. Detail information is listed in Supplementary Table 4. Hence, the results should be carefully interpreted with regard to the brain regions which did not survive at voxel-level *p* < 0.001. In addition, although the participants were carefully instructed before the experiment and asked about their states during post-experiment scans, we could not ensure the sleep–wake state of the subjects during the resting-state scan without fMRI-compatible EEG. Thus, future studies have to adopt certain measures to monitor the state of the subjects.

## Data Availability Statement

The raw data supporting the conclusions of this article will be made available by the authors, without undue reservation.

## Ethics Statement

The studies involving human participants were reviewed and approved by the Ethics Committee of the Henan Provincial People’s Hospital. The patients/participants provided their written informed consent to participate in this study.

## Author Contributions

XL, ZL, ZZ, and XW conceived the study, analyzed the data, and wrote the manuscript. HG, CW, JZ, FY, and SD designed and performed the experiments. FQ, MZ, JH, XQ, and HZ collected the clinical samples and performed the experiments. LT and YL conceived the study, designed the experiments, supervised the project, and wrote the manuscript. All authors contributed to the article and approved the submitted version.

## Conflict of Interest

The authors declare that the research was conducted in the absence of any commercial or financial relationships that could be construed as a potential conflict of interest.

## Publisher’s Note

All claims expressed in this article are solely those of the authors and do not necessarily represent those of their affiliated organizations, or those of the publisher, the editors and the reviewers. Any product that may be evaluated in this article, or claim that may be made by its manufacturer, is not guaranteed or endorsed by the publisher.
