# Supplementary material for: Real-Time fMRI Neurofeedback Training Changes Brain Degree Centrality and Improves Sleep in Chronic Insomnia Disorder: A Resting-State fMRI Study
Source: Front Mol Neurosci. 2022 Feb 23;15:825286. doi: 10.3389/fnmol.2022.825286 (PMC8904428; doi:10.3389/fnmol.2022.825286)
Supplement: Supplementary file 1 [file Data_Sheet_1.docx]

**Supplementary Figure**

**Figure S1** Brain areas that exhibited altered weighted DC induced by real-time fMRI neurofeedback training using different cut off thresholds (*r* = 0.15, 0.20, 0.25, 0.30, 0.35). Results were set at voxel-level *p* < 0.01, cluster-level *p* < 0.05, and *t* = 2.77 (Gaussian random field corrected). Warm colors indicate regions in which DC remarkably increased significantly, whereas cool colors indicate regions in which DC remarkably decreased significantly. The color bar indicates the *t*-value. Abbreviations:DC, degree centrality; L, left; R, right.


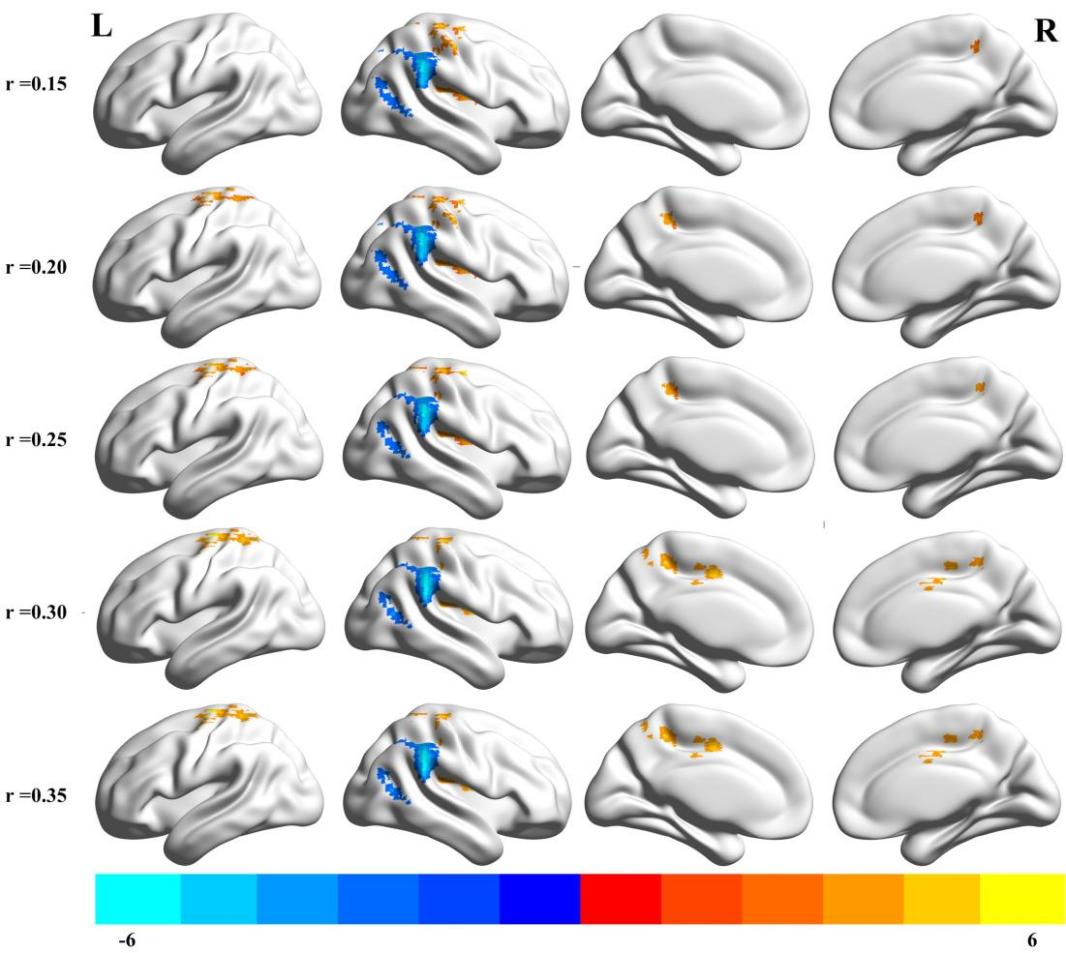


**Table S1**. Brain regions exhibited altered DC at different *r* threshold (post-trainging VS pre-training)

| **DC threshold** | **Brain regions** | **Whole cluster size** | **Cluster size** | **MNI coordinates** | | | ***t* score** |
| --- | --- | --- | --- | --- | --- | --- | --- |
|  |  |  |  | x | y | z |  |
| *r* = 0.15 | R Rolandic operculum | 212 | 52 | 48 | -24 | 21 | 5.589 |
|  | R Insula |  | 35 | 33 | -9 | 15 | 5.162 |
|  | R Putamen |  | 22 | 30 | -12 | 12 | 4.640 |
|  | R Supramarginal gyrus | 502 | 136 | 60 | -42 | 36 | -6.707 |
|  | R Angular gyrus |  | 86 | 33 | -57 | 42 | -4.711 |
|  | R Inferior parietal gyrus |  | 79 | 36 | -54 | 42 | -4.352 |
|  | R Middle occipital gyrus |  | 62 | 33 | -75 | 30 | -3.839 |
|  | R Middle temporal gyrus |  | 40 | 57 | -54 | 3 | -3.788 |
| *r* = 0.2 | R Postcentral gyrus | 382 | 76 | 51 | -18 | 51 | 4.255 |
|  | R Rolandic operculum |  | 59 | 48 | -21 | 21 | 5.829 |
|  | R Insula |  | 41 | 33 | -6 | 12 | 5.229 |
|  | R Putamen |  | 22 | 30 | -12 | 12 | 4.804 |
|  | R Supramarginal gyrus | 532 | 152 | 60 | -42 | 36 | -7.030 |
|  | R Inferior parietal gyrus |  | 95 | 51 | -42 | 45 | -4.531 |
|  | R Angular gyrus |  | 84 | 33 | -54 | 42 | -4.833 |
|  | R Middle occipital gyrus |  | 61 | 33 | -75 | 30 | -3.791 |
|  | R Middle temporal gyrus |  | 45 | 57 | -54 | 3 | -3.632 |
| *r* = 0.3 | B Postcentral gyrus | 717 | 158 | 39 | -30 | 42 | 4.155 |
|  | L Precental gyrus |  | 67 | -24 | -21 | 69 | 4.519 |
|  | R Rolandic operculum |  | 65 | 48 | -21 | 21 | 5.506 |
|  | B Superior parietal gyrus |  | 64 | -21 | -48 | 60 | 4.885 |
|  | R Insula |  | 40 | 33 | -6 | 12 | 5.320 |
|  | L Median cingulate gyrus |  | 31 | -6 | -39 | 48 | 3.805 |
|  | R Precuneus |  | 20 | 3 | -42 | 48 | 3.440 |
|  | R Supramarginal gyrus | 488 | 162 | 60 | -42 | 39 | -7.114 |
|  | R Inferior parietal gyrus |  | 92 | 51 | -42 | 45 | -4.978 |
|  | R Angular gyrus |  | 71 | 33 | -54 | 42 | -4.760 |
|  | R Middle occipital gyrus |  | 48 | 42 | -72 | 24 | -3.524 |
|  | R Middle temporal gyrus |  | 43 | 48 | -72 | 21 | -3.487 |
| *r* = 0.35 | B Postcentral gyrus | 1134 | 190 | -21 | -48 | 57 | 4.496 |
|  | B Median cingulate gyrus |  | 190 | 9 | -9 | 36 | 4.434 |
|  | L Precental gyrus |  | 82 | -24 | -18 | 66 | 4.762 |
|  | R Rolandic operculum |  | 68 | 48 | -21 | 21 | 4.993 |
|  | B Superior parietal gyrus |  | 76 | -21 | -48 | 60 | 5.230 |
|  | R Precuneus |  | 67 | 3 | -42 | 48 | 3.521 |
|  | R Insula |  | 40 | 33 | -6 | 12 | 5.145 |
|  | R Paracentral lobule |  | 28 | 15 | -39 | 51 | 3.723 |
|  | R Supramarginal gyrus | 350 | 158 | 60 | -42 | 39 | -7.026 |
|  | R Inferior parietal gyrus |  | 82 | 51 | -42 | 45 | -4.964 |
|  | R Angular gyrus |  | 57 | 33 | -54 | 42 | -4.524 |

Results were set at voxel-level: *p* < 0.01 , cluster-level: *p* < 0.05, *t* = 2.77 (Gaussian random field corrected). Abbreviations: DC, Degree Centrality; L, left; R, right;B, bilateral; MNI, Montreal Neurological Institute.

**Table S2** Brain regions that exhibited increased functional connectivity between post-training and pre-training revealed using seed-based method.

| **Seed regions** | **Brain regions** | **Abbreviations** | **Whole**  **Cluster size** | **Cluster size** | **MNI coordinates**  **of Cluster Peak** | | | ***t* score** |
| --- | --- | --- | --- | --- | --- | --- | --- | --- |
|  |  |  |  |  | x | y | z |  |
| R Postcentral gyrus | B Superior parietal gyrus | SPG | 1962 | 409 | 21 | -60 | 57 | 6.747 |
|  | B Precuneus | PCUN |  | 247 |  |  |  |  |
|  | B Superior occipital gyrus | SOG |  | 244 |  |  |  |  |
|  | B Postcentral gyrus | PoCG |  | 223 |  |  |  |  |
|  | B Cuneus | CUN |  | 171 |  |  |  |  |
|  | B Inferior parietal gyrus | IPG |  | 169 |  |  |  |  |
|  | B Middle occipital gyrus | MOG |  | 68 |  |  |  |  |
|  | R Precentral gyrus | PreCG |  | 67 |  |  |  |  |
|  | R Superior frontal gyrus, dorsolateral | SFGdor |  | 46 |  |  |  |  |
|  | R Supramarginal gyrus | SMG |  | 31 |  |  |  |  |
| R Rolandic operculum | B Lingual | LING | 2556 | 382 | 24 | -81 | -24 | 6.284 |
|  | B Calcarine | CAL |  | 244 |  |  |  |  |
|  | B Inferior occipital gyrus | IOG |  | 231 |  |  |  |  |
|  | L Fusiform | FFG |  | 202 |  |  |  |  |
|  | L Middle occipital gyrus | MOG |  | 81 |  |  |  |  |
|  | R Inferior temporal gyrus | ITG |  | 22 |  |  |  |  |
|  | R Inferior frontal gyrus, triangular part | IFGtriang | 556 | 156 | 54 | 18 | -6 | 5.272 |
|  | R Inferior frontal gyrus, orbital part | ORBinf |  | 110 |  |  |  |  |
|  | B Inferior frontal gyrus, opercular part | IFGoperc |  | 77 |  |  |  |  |
|  | R Middle frontal gyrus | MFG |  | 55 |  |  |  |  |
|  | R Insula | INS |  | 36 |  |  |  |  |
|  | R Superior temporal gyrus | STG |  | 30 |  |  |  |  |
|  | R Superior frontal gyrus, dorsolateral | SFGdor |  | 24 |  |  |  |  |
|  | R Precental gyrus | PreCG |  | 21 |  |  |  |  |
|  | B Median cingulate gyrus | MCG | 1332 | 335 | 42 | 0 | 51 | 5.036 |
|  | L Superior parietal gyrus | SPG |  | 148 |  |  |  |  |
|  | B Precuneus | PCUN |  | 114 |  |  |  |  |
|  | R Precental gyrus | PreCG |  | 112 |  |  |  |  |
|  | L Supplementary motor area | SMA |  | 82 |  |  |  |  |
|  | L Posterior cingulate gyrus | PCG |  | 44 |  |  |  |  |
|  | L Anterior cingulate gyrus | ACG |  | 40 |  |  |  |  |
|  | R Postcentral gyrus | PoCG |  | 39 |  |  |  |  |
|  | R Middle frontal gyrus | MFG |  | 31 |  |  |  |  |
|  | R Superior frontal gyrus, dorsolateral | SFGdor |  | 30 |  |  |  |  |
| R Insula | R Precental gyrus | PreCG |  | 130 | 33 | -6 | 15 | 5.615 |
|  | R Superior temporal gyrus | STG |  | 74 |  |  |  |  |
|  | R Insula | INS |  | 65 |  |  |  |  |
|  | R Temporal pole:superior temporal gyrus | TPOsup |  | 62 |  |  |  |  |
|  | R Rolandic operculum | ROL |  | 45 |  |  |  |  |
|  | R Inferior frontal gyrus, orbital part | ORBinf |  | 29 |  |  |  |  |
|  | B Fusiform | FFG | 2381 | 270 | 39 | -39 | -21 | 5.145 |
|  | B Lingual | LING |  | 325 |  |  |  |  |
|  | B Superior occipital gyrus | SOG |  | 238 |  |  |  |  |
|  | B Middle occipital gyrus | MOG |  | 234 |  |  |  |  |
|  | B Precuneus | PCUN |  | 184 |  |  |  |  |
|  | B Cuneus | CUN |  | 133 |  |  |  |  |
|  | L Superior parietal gyrus | SPG |  | 125 |  |  |  |  |
|  | B Calcarine | CAL |  | 117 |  |  |  |  |
|  | R Inferior temporal gyrus | ITG |  | 34 |  |  |  |  |
|  | L Posterior cingulate gyrus | PCG |  | 32 |  |  |  |  |
|  | L Inferior parietal gyrus | IPG |  | 31 |  |  |  |  |
|  | L Median cingulate gyrus | MCG |  | 26 |  |  |  |  |
|  | R Inferior occipital gyrus | IOG |  | 24 |  |  |  |  |
| R Superior parietal gyrus | R Postcentral gyrus | PoCG | 1252 | 429 | 36 | -36 | 48 | 6.555 |
|  | R Precental gyrus | PreCG |  | 166 |  |  |  |  |
|  | R Supramarginal gyrus | SMG |  | 107 |  |  |  |  |
|  | R Superior parietal gyrus | SPG |  | 94 |  |  |  |  |
|  | R Inferior parietal gyrus | IPG |  | 73 |  |  |  |  |
|  | R Superior frontal gyrus, dorsolateral | SFGdor |  | 34 |  |  |  |  |
|  | R Rolandic operculum | ROL |  | 32 |  |  |  |  |
|  | R Precuneus | PCUN |  | 22 |  |  |  |  |
|  | L Postcentral gyrus | PoCG | 922 | 308 | -30 | -24 | 57 | 5.270 |
|  | L Inferior parietal gyrus | IPG |  | 230 |  |  |  |  |
|  | L Precental gyrus | PreCG |  | 178 |  |  |  |  |
|  | L Supramarginal gyrus | SMG |  | 80 |  |  |  |  |
|  | L Superior parietal gyrus | SPG |  | 69 |  |  |  |  |

Results were set at voxel-level: *p* < 0.01 , cluster-level: *p* < 0.05, *t* = 2.77 (Gaussian random field corrected). Abbreviations: DC, Degree Centrality; L, left; R, right;B, bilateral; MNI, Montreal Neurological Institute.

**Table S3** Brain regions that exhibited decreased functional connectivity between post-training and pre-training revealed using seed-based method.

| **Seed regions** | **Brain regions** | **Abbreviations** | **Whole**  **Cluster size** | **Cluster size** | **MNI coordinates** | | | ***t* score** |
| --- | --- | --- | --- | --- | --- | --- | --- | --- |
|  |  |  |  |  | x | y | z |  |
| R Supramarginal gyrus | R Middle frontal gyrus | MFG | 602 | 280 | 42 | 45 | 15 | -5.689 |
|  | R Inferior frontal gyrus, triangular part | IFGtriang |  | 145 |  |  |  |  |
|  | R Inferior frontal gyrus, opercular part | IFGoperc |  | 80 |  |  |  |  |
|  | R Middle frontal gyrus, orbital part | ORBmid |  | 34 |  |  |  |  |
|  | L Inferior frontal gyrus, triangular part | IFGtriang | 414 | 137 | -45 | 45 | 12 | -5.051 |
|  | L Middle frontal gyrus | MFG |  | 88 |  |  |  |  |
|  | L Inferior frontal gyrus, orbital part | ORBinf |  | 77 |  |  |  |  |
|  | L Insula | INS |  | 37 |  |  |  |  |
|  | L Middle frontal gyrus, orbital part | ORBmid |  | 20 |  |  |  |  |
| R Inferior parietal gyrus | R Supramarginal gyrus | SMG | 265 | 181 | 57 | -39 | 27 | -5.367 |
|  | R Superior temporal gyrus | STG |  | 46 |  |  |  |  |
|  | R Angular | ANG |  | 25 |  |  |  |  |
| R Angular | L Cuneus | CUN | 166 | 68 | -21 | 66 | 33 | -4.483 |
|  | L Superior occipital gyrus | SOG |  | 47 |  |  |  |  |

Results were set at voxel-level:*p* < 0.01 , cluster-level: *p* < 0.05, *t* = 2.77 (Gaussian random field corrected). Abbreviations: DC, Degree Centrality; L, left; R, right;B, bilateral; MNI, Montreal Neurological Institute.

**Table S4** Brain regions exhibited altered DC at *r* = 0.25 (post-trainging VS pre-training)

| Brain regions | Whole  Cluster size | Cluster size | MNI coordinates | | | t score |
| --- | --- | --- | --- | --- | --- | --- |
|  |  |  | x | y | z |  |
| R Rolandic operculum | 102 | 29 | 48 | -21 | 21 | 5.865 |
| R Insula |  | 29 | 33 | -6 | 12 | 5.388 |
| R Supramarginal gyrus | 128 | 103 | 60 | -42 | 36 | -7.105 |
| R Inferior parietal gyrus |  | 121 | 51 | -42 | 45 | -4.831 |

Results were set at voxel-level: *p* < 0.001 , cluster-level: *p* < 0.05, *t* = 3.29 (GRF corrected). Abbreviations: DC, Degree Centrality; R, right; MNI, Montreal Neurological Institute.
